# Incidental Diagnosis of Monomorphic Epitheliotropic Intestinal T-Cell Lymphoma: A Case Report

**DOI:** 10.7759/cureus.10084

**Published:** 2020-08-27

**Authors:** Harish Gopalakrishna, Ahmad Al-abdouh, Gayatri Nair, Ammer Bekele

**Affiliations:** 1 Internal Medicine, Saint Agnes Hospital, Baltimore, USA

**Keywords:** monomorphic epitheliotropic intestinal t-cell lymphoma, intestinal t-cell lymphoma, type ii enteropathy associated t-cell lymphoma, meitl

## Abstract

Monomorphic epitheliotropic intestinal T-cell lymphoma (MEITL) is a rare, rapidly progressive, primary intestinal T-cell lymphoma. It is most commonly seen in the Asian and Hispanic populations and is usually not related to celiac disease, unlike type I enteropathy associated T-cell lymphoma. The most common site of occurrence is the small intestine. Patients usually present during the advanced stage of disease with clinical features of intestinal perforation or obstruction. The late clinical presentation and lack of targeted therapy are factors contributing to its poor prognosis. Here, we are presenting the case of a patient who initially came to the hospital for a urinary tract infection. As his abdominal CT scan showed abdominal wall thickening, he underwent further workup which revealed the diagnosis of MEITL.

## Introduction

Monomorphic epitheliotropic intestinal T-cell lymphoma (MEITL) is a primary intestinal T-cell lymphoma. It was formerly known as type II enteropathy associated T-cell lymphoma. It is different from type I enteropathy associated T-cell lymphoma because of its distinctive nature and lack of association with celiac disease [1]. It has been described as more common in the Asian and rare in the western population. It occurs over a wide age range with a median age of 58 years and with a male to female ratio of 2:1 [2]. The most common presentation is intestinal perforation or obstruction, and this was described in more than half of the patients. Other less common presenting symptoms include diarrhea or weight loss [1]. The most common site of involvement is the small intestine especially the jejunum with some cases reported in the stomach as well [2]. We are reporting a case of an unfortunate 53-year-old Caucasian gentleman who initially presented for urinary tract infection and was incidentally found to have abdominal wall thickening on the CT scan which turned out to be MEITL.

## Case presentation

A 53-year-old gentleman with a history of intellectual disability and no other significant past medical or surgical history came with complaints of diffuse achy abdominal pain of two-week duration. The intensity of pain was described as 5-6 on a scale of 1 to 10, with location more on the right lower quadrant. He also complained of associated fever, burning sensation with micturition and increased urinary frequency. He also reported abdominal distention and constipation of two days duration but was able to pass gas. He denied smoking, use of alcohol or any illicit drugs. His family history was significant for colon cancer in his father.

On initial examination, the temperature was 38.3℃, heart rate 103 per minute, blood pressure 114/72 mmHg, and oxygen saturation of 98% on room air. He had normal heart sounds and clear air entry bilaterally on lung auscultation. Abdominal examination was significant for hyperactive bowel sounds, distension, and diffuse tenderness with voluntary guarding. There was no costovertebral angle tenderness. No rashes were seen upon skin examination.

Laboratory findings on admission revealed the following: hemoglobin 9.5 g/dL (normal range = 13-17 g/dL); mean corpuscular volume 84.5 fL (normal range 80-99 fL); white blood cells (WBCs) 30.1 K/microliter (normal range = 4-11 K/microliter) with 95% neutrophils (normal range = 35%-70%), band neutrophils 1% (normal range = 0%-5%) and lymphocytes 1% (normal range = 20%-45%). Peripheral smear analysis showed moderate granulocytosis, mature leukocytes without dyspoiesis and normocytic anemia. Iron panel showed: iron 18 ug/dL (normal range = 60-160 ug/dL), total iron binding capacity 147 ug/dL (normal range = 250-450 ug/dL), transferrin saturation 12% (normal range = 14%-50%) and ferritin 1324 ng/mL (normal range = 30-400 ng/mL). Additional workup done included carcinoembryonic antigen 0.9 ng/mL (normal range = 0-3.4 ng/mL), lactate dehydrogenase 159 U/L (normal range = 135-225 U/L) and uric acid 3.8 mg/dL (normal range < 7.1 mg/dL). Urine analysis showed presence of leukocyte esterase and WBC of 21-50/hpf (normal range = 0-2/hpf). Patient tested negative for HIV 1 and 2.

**Figure 1:**
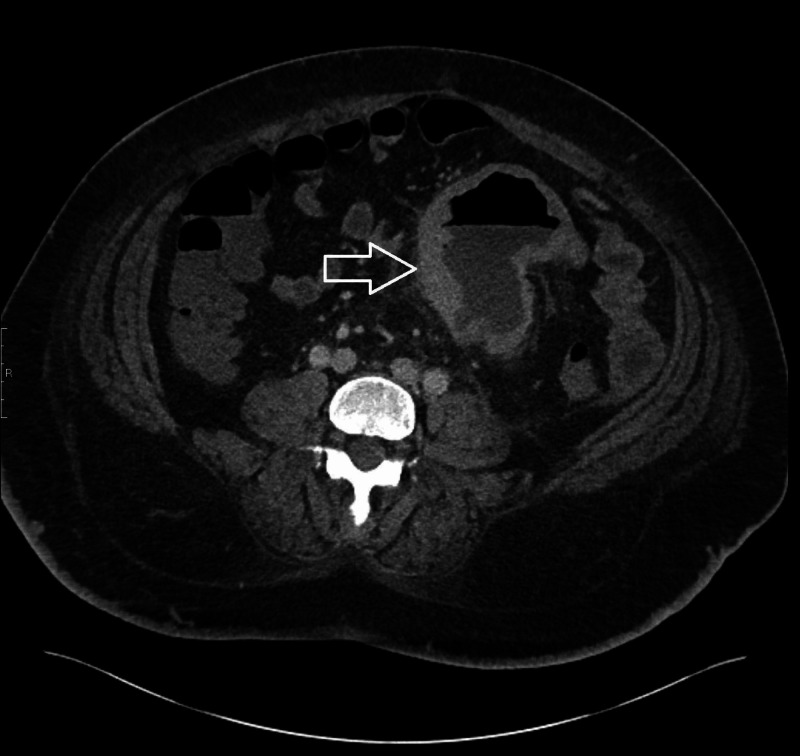
CT scan of the abdomen and pelvis: transversal view

**Figure 2:**
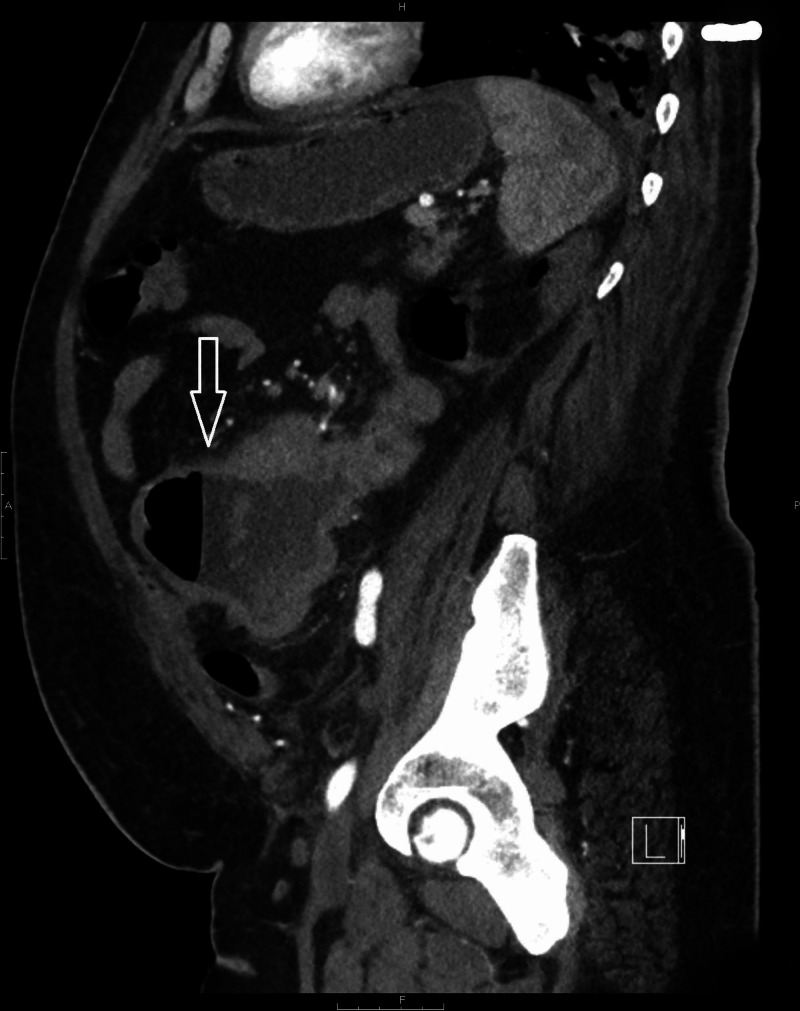
CT scan of the abdomen and pelvis: sagittal view

**Figure 3:**
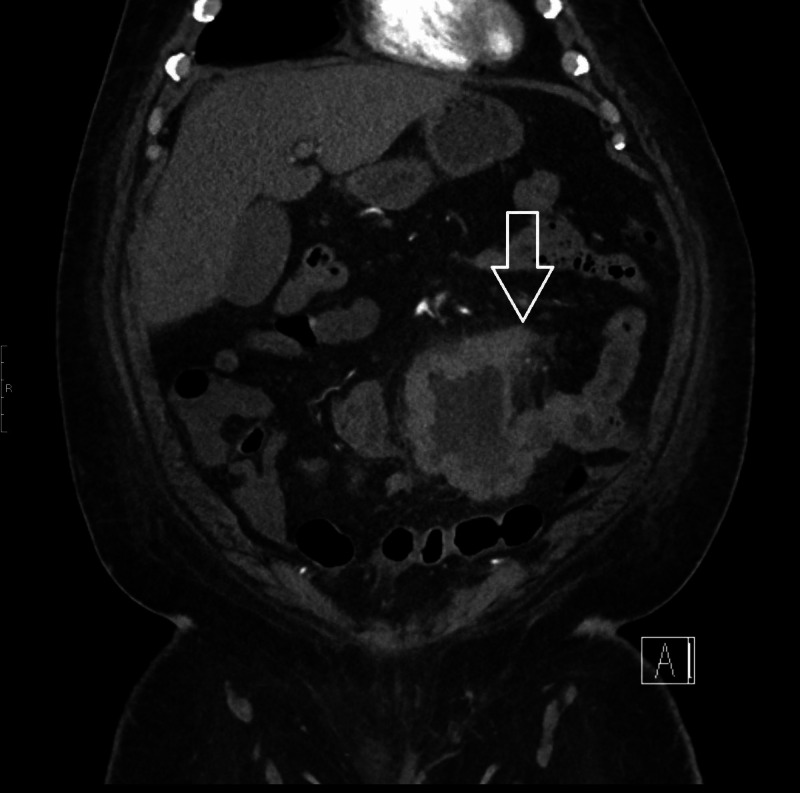
CT scan of the abdomen and pelvis: coronal view

**Figure 4:**
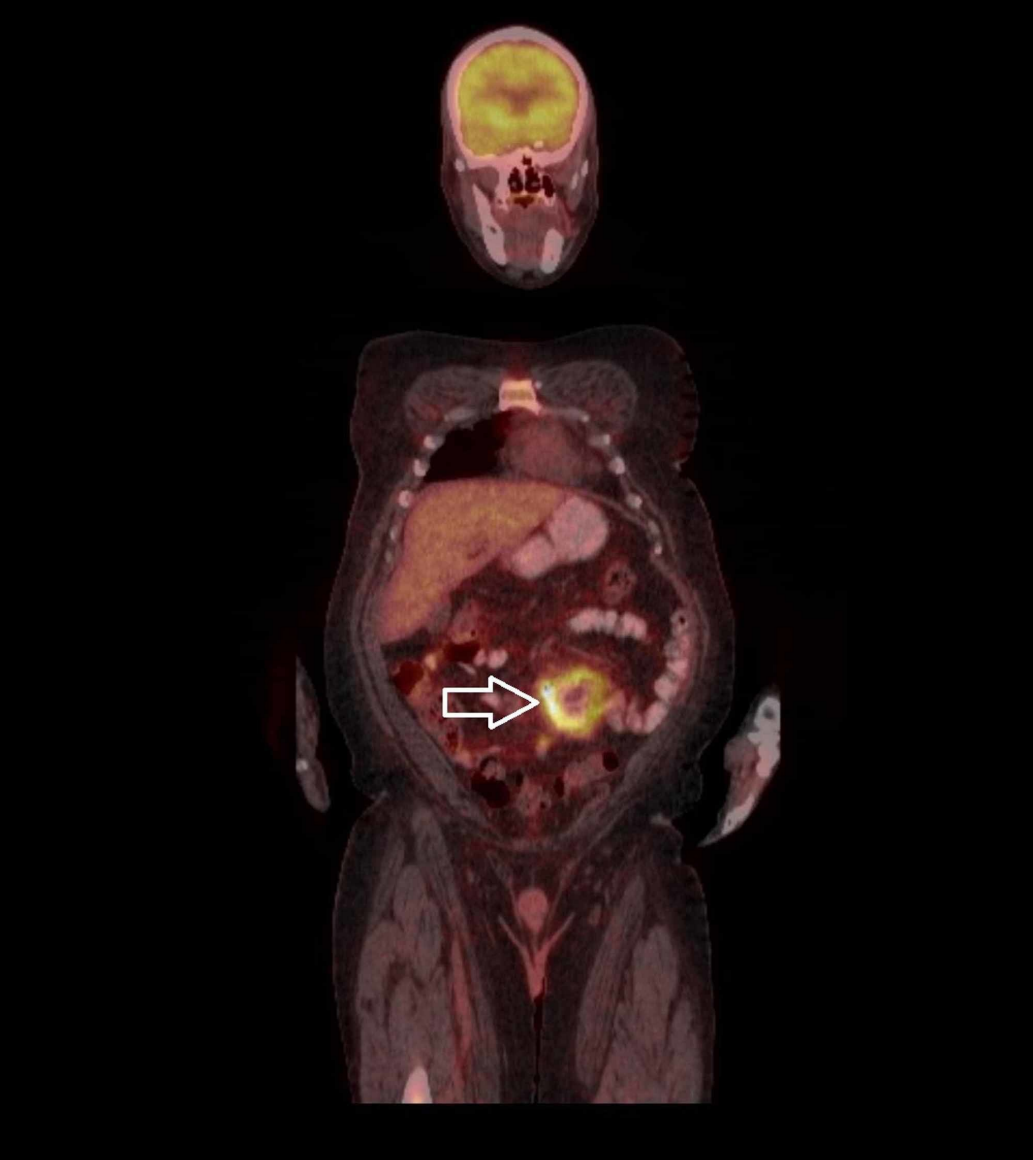
Whole body PET-CT PET-CT, positron emission tomography-computed tomography

CT scan of the abdomen and pelvis showed congenital malposition of left kidney with surrounding perinephric stranding suggestive of pyelonephritis. It also showed a thick-walled prominent dilated loop of small bowel seen within the left side of the abdomen with an air-fluid level and surrounding inflammatory changes (Figures 1, 2, 3). He also had a whole-body positron emission tomography-computed tomography (PET-CT) which showed metabolically active wall thickening in the proximal jejunum along with hypermetabolic conglomerate pelvic lymphadenopathy anterior to the lumbosacral spine compatible with metastatic intestinal lymphoma (Figure 4). 

He was treated with ceftriaxone for management of pyelonephritis and his symptoms improved.

**Figure 5:**
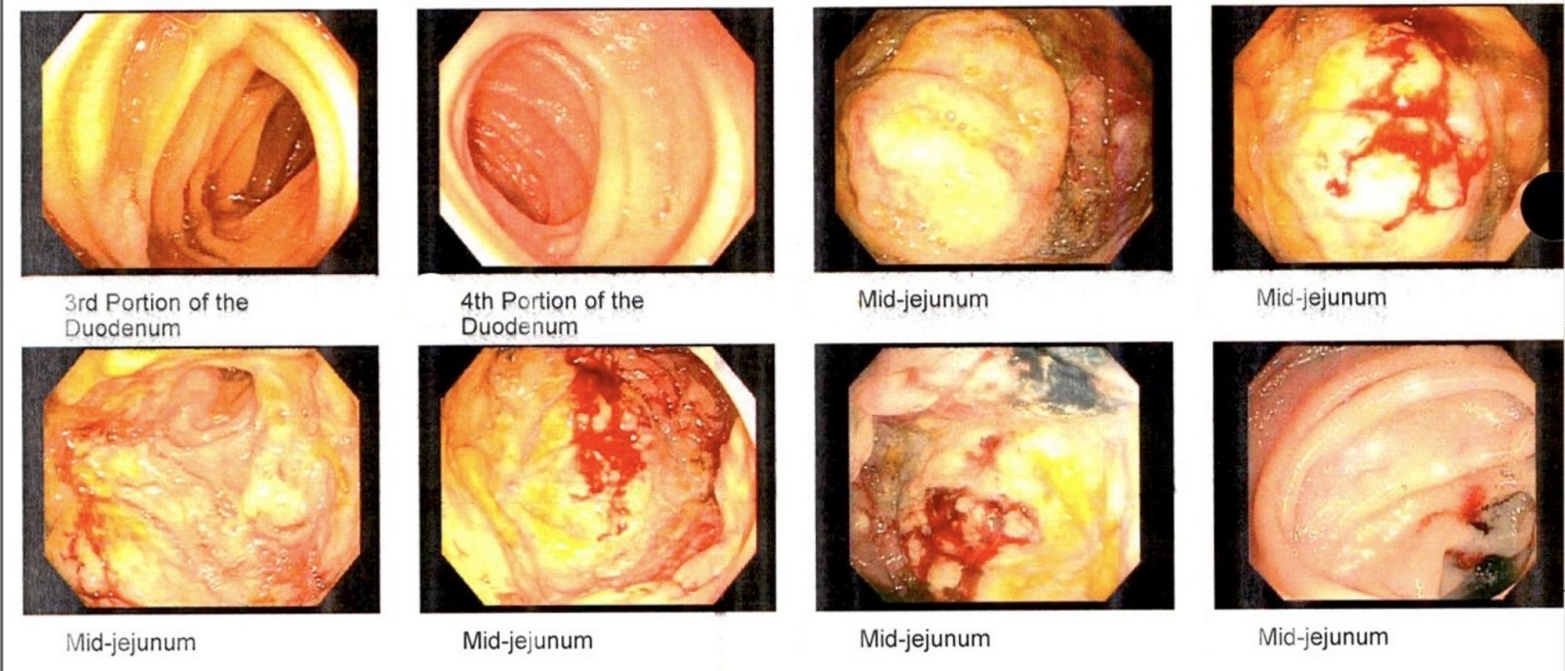
Enteroscopy pictures showing the mass

Given the high suspicion for malignancy, he underwent a small bowel enteroscopy which showed a large fungating and ulcerated mass in mid-jejunum which was biopsied (Figure 5). He also had screening colonoscopy and endoscopy which were unremarkable.

**Figure 6:**
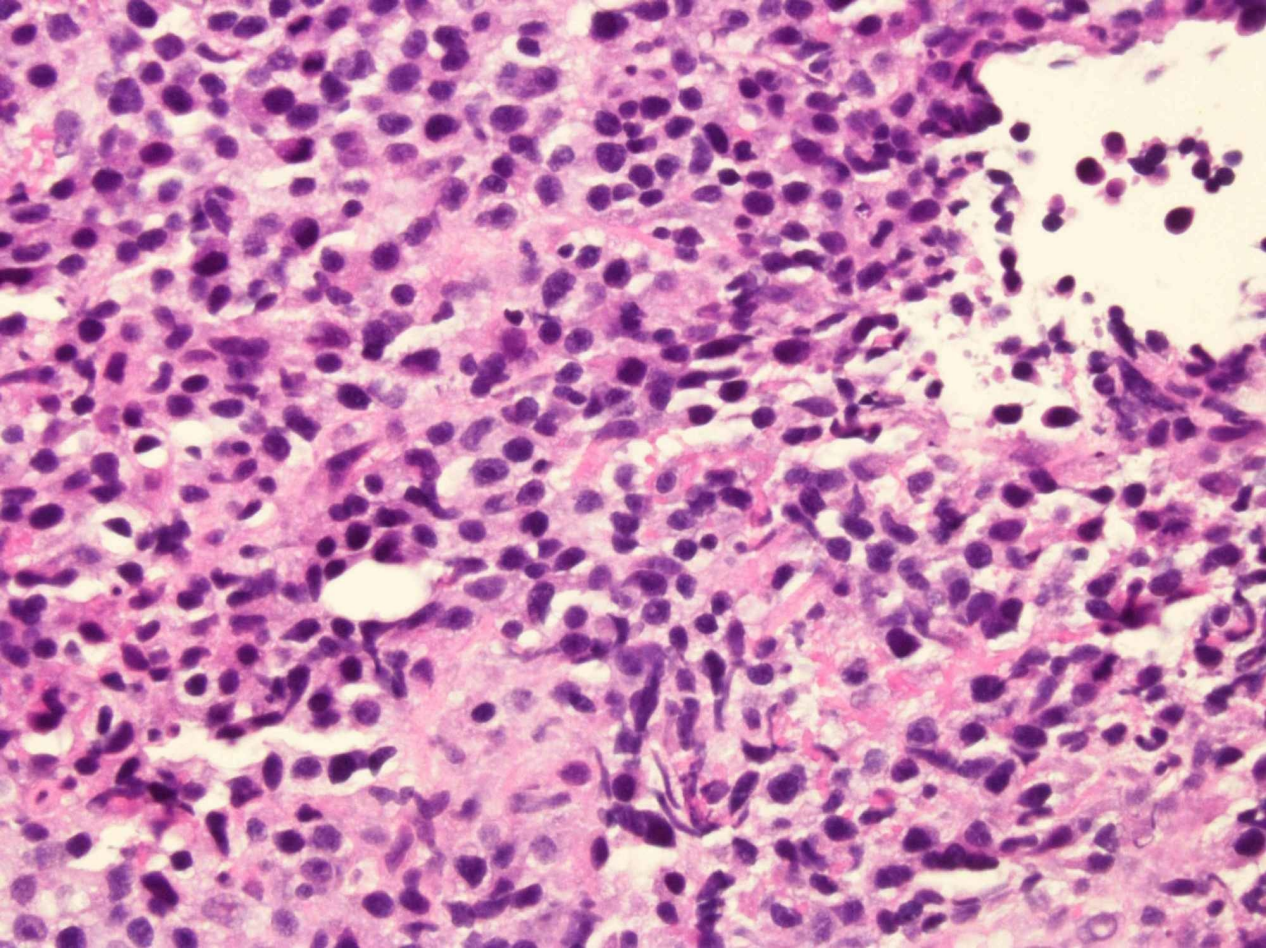
Light microscopy of the biopsied mass

**Figure 7:**
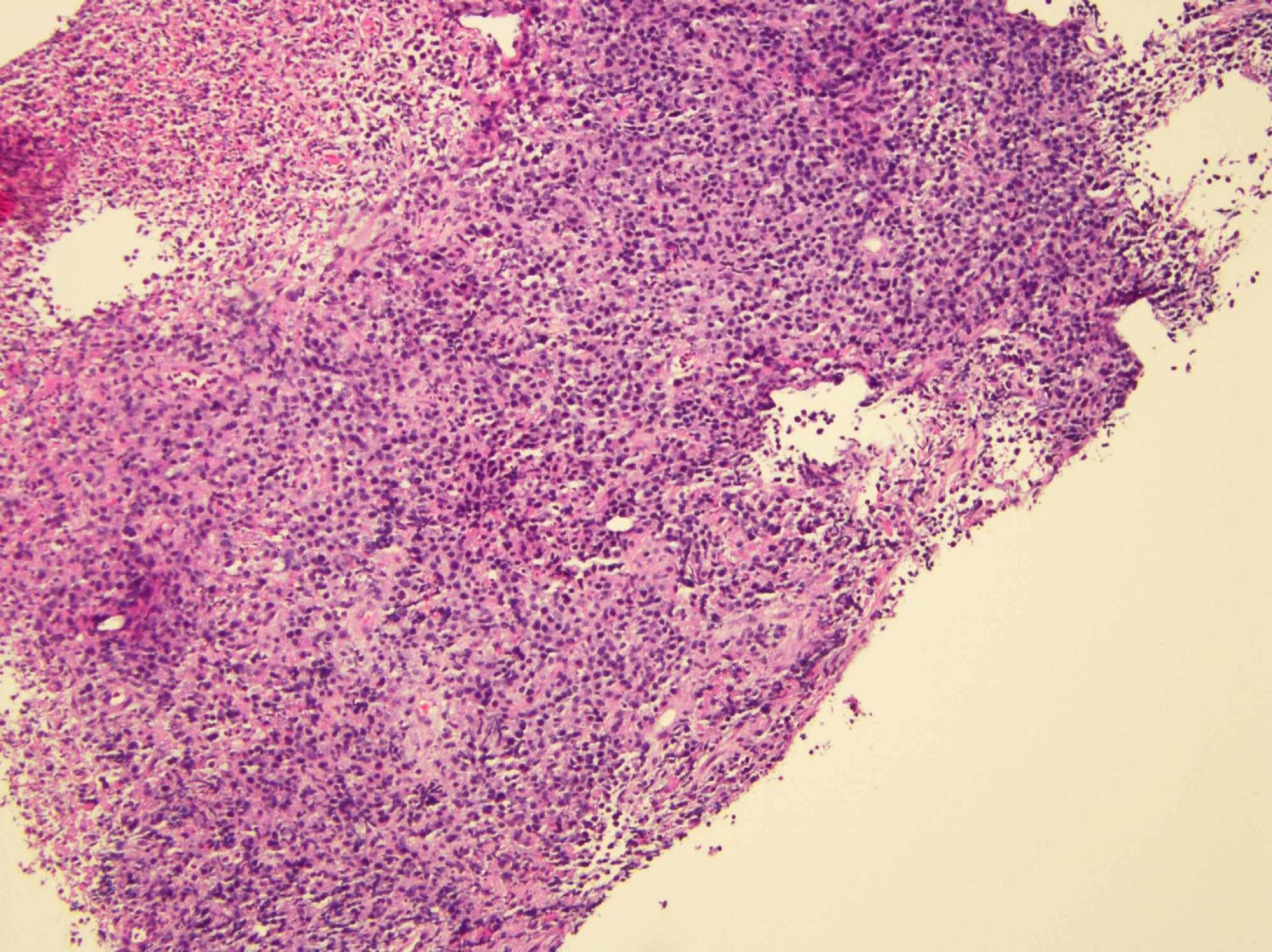
Light microscopy of the biopsied mass

Jejunal mass biopsy pathology showed ulcerated tissue without intact epithelium composed of sheets of atypical medium-sized mononuclear cells with pale cytoplasm, dispersed chromatin and visible nucleoli (Figures 6, 7). Immunohistochemical stains were positive for CD45, CD3, CD 56, CD8, CD 7 and TIA-1 and negative for CD5, CD20, CD30, pancytokeratin, synaptophysin, chromogranin KI-67. These results were suggestive of MEITL.

Given the high risk for tumor lysis syndrome and intestinal perforation, he was started on chemotherapy with cyclophosphamide, doxorubicin, vincristine, etoposide and prednisone (CHOEP) over three days in the hospital after obtaining baseline echocardiography and chest port placement. He received three doses of the treatment and was discharged with advice to have close follow-up with an oncologist. On follow up he was readmitted to the hospital two weeks after his first cycle of chemotherapy for febrile neutropenia which was treated initially with cefepime for five days and was discharged on trimethoprim /sulfamethoxazole and levofloxacin to complete a 10-day course of antibiotics.

## Discussion

MEITL is a primary intestinal T-cell lymphoma caused due to malignant proliferation of intraepithelial lymphocytes [1]. It is a rare tumor that accounts for less than 5% of all gastrointestinal lymphomas and less than 1% of all non-Hodgkin's lymphomas [3]. It was previously classified under enteropathy-associated T-cell lymphoma (EATL). Before 2016, EATL was classified into classic or type I EATL and simplex or type II EATL. Type I EATL is seen commonly in northern European descent and is associated with celiac disease. Since there is a profound variation from type 1, type II has been classified as a separate entity called MEITL [1]. 

Most cases of MEITL are derived from γδ T cells, with certain exceptions like T-cell receptor (TCR) silent and some cases express TCR αβ [1]. MEITL is an intestinal lymphoma with monomorphic small to medium-sized cells with minimal pleomorphism. Majority of lymphoma cells are CD2+, CD3+, CD7+, CD8+, CD56+, CD4-, CD5- and CD 30- [2]. This patient was positive for CD 3, CD 7, CD 8, CD 45 and negative for CD 5 and CD 30, making MEITL the most likely diagnosis. There are exceptions to this rule with cases of CD 8- and CD 56- MEITL lymphoma described [2]. Usually, patients present with intestinal perforation, obstruction or vague abdominal complaints. Common sites of involvement are jejunum as in our patient followed by ileum and duodenum, rarely involving a large bowel and stomach. It is common to metastasize to mesenteric lymph nodes, but can also spread to lungs, liver and central nervous system [2]. Most of the cases also expressed cytotoxic marker TIA [1]. Most are positive for megakaryocyte associated tyrosine kinase. There have been studies that showed spleen tyrosine kinase (SYK) expression in 95% of MEITL cases [2]. STAT5B mutations were reported in 36% of cases of MEITL, and all of which were of γδ T-cell origin [4]. STAT5B mutations are very uncommon and are associated with more clinically aggressive disease [5]. Even though MEITL is not commonly associated with celiac disease, there are cases of MEITL seen in patients with histologically proven celiac disease [6]. 

There is no specific treatment for MEITL and treatment options such as chemotherapy, surgery or radiotherapy alone, have not been proven to improve the outcome. Although the combination of stem cell transplant with other treatment modalities have been shown to improve outcome in EATL, this cannot be strictly applied to MEITL because of clinical variation in disease entity [7,8]. Current guidelines suggest a systemic anthracycline-containing chemotherapy with primary surgical resection to be an effective regimen [7]. A regimen containing cyclophosphamide, doxorubicin, vincristine and prednisone (CHOP) is the most frequently used chemotherapy [7]. Our patient received a modified regimen which included etoposide along with CHOP regimen. The prognosis of MEITL is poor with a reported median survival of only seven months and one-year overall survival is only 36% [2]. This is mostly due to late presentation and lack of targeted therapy. 

This case was a MEITL masquerading as sepsis. In spite of appropriate improvement with antibiotics, the concern for a possible underlying malignancy on imaging studies led to extensive workup and timely diagnosis with an early intervention which proved beneficial for the patient.

## Conclusions

MEITL is a rare and aggressive disease with poor prognosis. Even though adequate information is known about the immunophenotype of the disease, there is a lack of targeted immunotherapy to improve clinical outcome. Lack of knowledge about predisposing factors also makes it a challenge to predict people at risk.
